# Identification of novel genes potentially involved in somatic embryogenesis in chicory (*Cichorium intybus *L.)

**DOI:** 10.1186/1471-2229-10-122

**Published:** 2010-06-22

**Authors:** Anca Lucau-Danila, Laurent Laborde, Sylvain Legrand, Ludovic Huot, David Hot, Yves Lemoine, Jean-Louis Hilbert, Simon Hawkins, Marie-Christine Quillet, Theo Hendriks, Anne-Sophie Blervacq

**Affiliations:** 1 UMR USTL-INRA 1281, Stress Abiotiques et Différenciation des Végétaux cultivés, Université Lille1, Cité Scientifique SN2, F-59650 Villeneuve d'Ascq, France; 2 Novartis Pharma AC, ONC/DD 11/BIO Lab MAIRA, Basel, Switzerland; 3 EA 3061, Biotechnologies Végétales appliquées aux Plantes Aromatiques et Médicinales, Université Jean Monnet, 23 rue du docteur Paul Michelon, F-42000, Saint-Etienne, France; 4 U1019, UMR8204, Transcriptomics and applied Genomics, Institut Pasteur de Lille, Center for Infection and Immunity of Lille (CIIL), 1 rue du professeur Calmette, F-59019 Lille, France

## Abstract

**Background:**

In our laboratory we use cultured chicory (*Cichorium intybus*) explants as a model to investigate cell reactivation and somatic embryogenesis and have produced 2 chicory genotypes (K59, C15) sharing a similar genetic background. K59 is a responsive genotype (embryogenic) capable of undergoing complete cell reactivation *i.e*. cell de- and re-differentiation leading to somatic embryogenesis (SE), whereas C15 is a non-responsive genotype (non-embryogenic) and is unable to undergo SE. Previous studies [1] showed that the use of the β-D-glucosyl Yariv reagent (β-GlcY) that specifically binds arabinogalactan-proteins (AGPs) blocked somatic embryo production in chicory root explants. This observation indicates that β-GlcY is a useful tool for investigating somatic embryogenesis (SE) in chicory. In addition, a putative AGP (DT212818) encoding gene was previously found to be significantly up-regulated in the embryogenic K59 chicory genotype as compared to the non-embryogenic C15 genotype suggesting that this AGP could be involved in chicory re-differentiation [2]. In order to improve our understanding of the molecular and cellular regulation underlying SE in chicory, we undertook a detailed cytological study of cell reactivation events in K59 and C15 genotypes, and used microarray profiling to compare gene expression in these 2 genotypes. In addition we also used β-GlcY to block SE in order to identify genes potentially involved in this process.

**Results:**

Microscopy confirmed that only the K59, but not the C15 genotype underwent complete cell reactivation leading to SE formation. β-GlcY-treatment of explants blocked *in vitro *SE induction, but not cell reactivation, and induced cell wall modifications. Microarray analyses revealed that 78 genes were differentially expressed between induced K59 and C15 genotypes. The expression profiles of 19 genes were modified by β-GlcY-treatment. Eight genes were both differentially expressed between K59 and C15 genotypes during SE induction and transcriptionally affected by β-GlcY-treatment: *AGP *(DT212818), *26 S proteasome AAA ATPase subunit 6 *(*RPT6*), *remorin *(*REM*), *metallothionein-1 *(*MT1*), two non-specific lipid transfer proteins genes (*SDI-9 and DEA1*), *3-hydroxy-3-methylglutaryl-CoA reductase *(*HMG-CoA reductase*), and *snakin 2 *(*SN2*). These results suggest that the 8 genes, including the previously-identified *AGP *gene (DT212818), could be involved in cell fate determination events leading to SE commitment in chicory.

**Conclusion:**

The use of two different chicory genotypes differing in their responsiveness to SE induction, together with β-GlcY-treatment represented an efficient tool to discriminate cell reactivation from the SE morphogenetic pathway. Such an approach, together with microarray analyses, permitted us to identify several putative key genes related to the SE morphogenetic pathway in chicory.

## Background

Plants show a high level of plasticity and adapt to changing environmental conditions by extensive modifications in developmental programmes. A striking example concerns the plant's capacity to implement cell pluripotency and totipotency programmes [3]. In pluripotency, a single cell gives rise to most, but not all, of the various cell types that make up a plant. In totipotency, a single cell can develop into an embryo (under certain conditions), thereby producing a new adult organism. During both of these programmes, a single differentiated somatic cell re-enters the cell cycle *via *the cell-reactivation process. Cell reactivation proceeds in two phases [3-5]. Some cells, dispersed within the mesophyll tissue of *in vitro *plantlets seem particularly responsive when induced in an appropriate culture medium. Once induced, these cells - referred to as 'competent' cells - become committed to different morphogenetic pathways such as organogenesis and callogenesis (pluripotency) or somatic embryogenesis (totipotency). However, not all competent cells are able to re-enter the cell cycle and undergo cellular division (even under appropriate culture conditions) and are referred to as 'reactivating cells' (RC). In contrast, if competent cells are able to undergo mitosis they are referred to as 'fully reactivated cells' (FRC).

In the context of our research into plant cell reactivation in chicory, we had previously used the interspecific hybrid '474' (*C. intybus *L. × *C. endivia *L.) [6,7]. In this model, somatic embryos can develop rapidly, in high numbers, and directly from single reactivated cells of different vegetative or reproductive explants (leaves, roots, styles, etc.) [1,4,8-13]. Previous studies have shown that the cell reactivation process takes place between d0 and d4 in *in vitro *cultures of the hybrid '474' [4,7]. Unfortunately, the interspecific hybrid '474' is unsuitable for genetic analyses because of its sterility, and two new experimental genotypes were, therefore, selected: i) a responsive genotype was identified as highly embryogenic in a hybrid population of the Hungarian cultivar Koospol (*C. intybus*) and labelled K59 and ii) a non-responsive genotype, labelled C15, was identified as nonembryogenic among the descendants produced by repeated selfing of the K59 genotype [2].

The generation of subtractive cDNA libraries (K59 *vs *C15 at d4) and sequencing of ESTs enabled our laboratory to obtain preliminary molecular information concerning differences in gene expression between the two genotypes [2]. This study showed that several genes were differentially expressed between the 2 genotypes during somatic embryogenesis and that a gene, encoding a potential arabinogalactan protein (AGP) (contig 0687), was significantly over-expressed in the embryogenic K59 genotype as compared to the non-embryogenic genotype C15. Such an observation is interesting as we had previously shown that the β-D 1,3 Glucosyl Yariv reagent (β-Glc Y) that specifically binds AGPs blocked somatic embryo production in chicory root explants [1]. These two results could suggest that AGPs are involved in cell reactivation in chicory. AGPs are a class of cell wall proteoglycans usually containing a hydroxyproline-rich core protein backbone linked to a very large carbohydrate moiety representing 90-98% of the molecular mass [14,15]. AGPs are widely distributed in higher plants [16] where they are believed to play multiple roles in vegetative-, reproductive-, and cellular-growth and development [17-19]. AGPs have also been associated with the cell re-differentiation process in both carrot and chicory [1,13,20-22].

β-Glc Y is a synthetic molecule that specifically interacts with AGPs in a non-covalent manner [23,24,18] and has been used to selectively precipitate AGPs *in muro *thereby interfering with their functional activity [25]. The use of β-Glc Y has been shown to inhibit cell growth [26], to alter cell elongation in *Arabidopsis *roots [27], to modify programmed cell death and differentiation [28], to inhibit pollen tube growth [29,30], and to inhibit both SE [1,31] and zygotic embryo development [32].

In this study we used 2 different genotypes (K59: responsive; C15: non-responsive) together with β-Glc Y-treatment in an original approach to discriminate cell reactivation from *in vitro *morphogenesis and to analyse cell events during SE induction in chicory. Transcriptomic analyses were performed on probes (annotated chicory ESTs) pre-selected by SSH. Microarrays representing 1,098 unique genes were used to investigate the expression profiles of the two chicory genotypes in the presence and absence of β-Glc Y.

## Results and discussion

### 1. Cytological characterization of cell reactivation (CR) events in chicory

#### The K59 but not C15 genotype shows complete cell reactivation (CR) and somatic embryogenesis (SE) in cultured leaves

**Figure 1:**
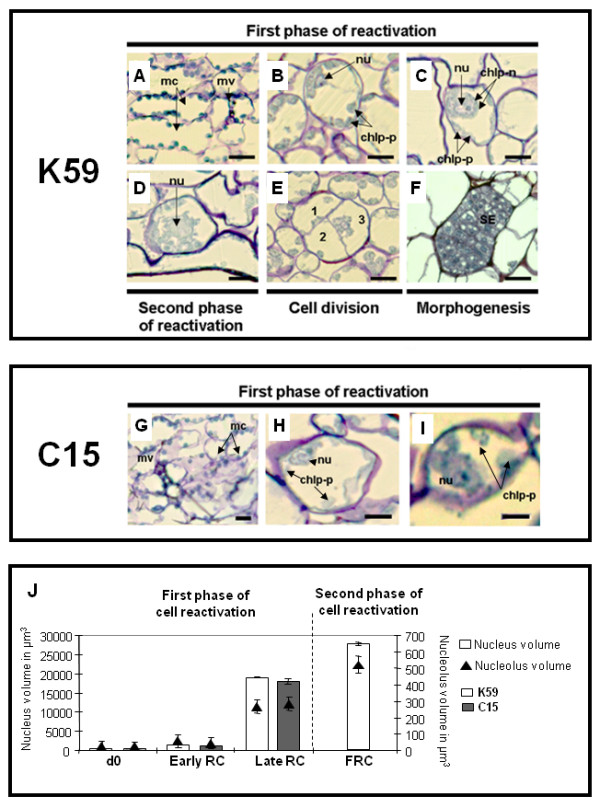
**Cell reactivation and somatic embryogenesis in leaf explants from two chicory genotypes**. A: Sections of K59 leaf explants from in vitro plantlets at d0. B: Early reactivating cells. C: Late reactivating cells. D: The second phase of the cell reactivation (d4). E: Cell divisions (d4). Daughter-cells (1, 2, 3) derived from mitosis of a single FRC. F: Morphogenesis (d11). Somatic embryos (SE) are detected as compact, rectangular-shaped and are composed of dense-embryonary cells. G: Sections of C15 leaf explants from in vitro plantlets at d0. The first phase of cell reactivation in C15 leaf explants proceeds in as similar way to that observed in K59: increase of nuclear volume (H), relocation of plastids around the voluminous nucleus and occurrence of a partial plastid crown (I). Neither fully reactivated cells nor cell division events can be observed. J: Evolution of nuclear- and nucleolar-volumes during cell reactivation in the K59 genotype. The first phase of cell reactivation is characterised by a significant increase in both nuclear volume (white bar, × 40 volume increase) and nucleolar volume (black triangle, × 67) volume increase) as compared to those of differentiated mesophyll cells. The second phase of cell reactivation is characterised by a slight increase of nuclear volume (white bar, × 1.5) and nucleolar volume (black triangle, × 1.9) as compared to the first phase of cell reactivation. Fully reactivated-cell status is given to those dedifferentiated cells able to re-enter the cell cycle in only K59. mc: differentiated mesophyll cells; mv: minor veins; RC: reactivating cells; nu: nucleus; chlp-p: chloroplasts in parietal position; chlp-n: perinuclear crown of chloroplasts; n = 10 semi-thin sections from K59 leaf explants. Bars represent standard error. A-L: Three micrometer-semi-thin sections stained with TBO. Bar = 20 μm.

We have previously reported preliminary cytological studies on cell reactivation in K59 and C15 chicory explants from greenhouse-grown plants [2]. Here, we present detailed cytological analyses of the two phases that occur during cell reactivation in explants obtained from *in vitro*-grown plantlets of these 2 genotypes (Fig. 1 and additional file 1).

The first phase of cell reactivation involved several stages of cell reorganisation. At d0, leaves from 6-week old plantlets (both genotypes) contained 5-6 layers of differentiated spongy mesophyll tissue but no palisade mesophyll bordered by a thin epidermis (Fig. 1A, G). Differentiated mesophyll cells were rectangular and characterized by the presence of a thin cell wall lined with a layer of cytoplasm, a turgid single vacuole and a small nucleus (often absent depending upon the section). In addition, numerous chloroplasts (14-16 per section) were observed adjacent to the cell wall. These chloroplasts were flattened and stained strongly with TBO suggesting the presence of high amounts of proteins. Observation of day 4 explants revealed that a small proportion of cells had started to undergo structural reorganisations associated with the first phase of cell reactivation (CR). This first phase can be divided into 2 stages (Early CR and Late CR). The first stage (Early CR) involved modifications to the nucleus that became more spherical and was therefore more easily distinguished in a section plane. In addition, a small nucleolus also appeared (Fig. 1B, H, J).

The next stage (Late CR) was characterised by a large increase in nuclear volume, as well as by important modifications in the structure and distribution of other cellular components (Fig. 1C, I, J). Chloroplasts became more spherical and stained less strongly with TBO. These organelles were located near to the cell wall (as previously observed in early CR) and were also observed next to the nucleus (Fig. 1C, I). In some cases plastids formed a perinuclear crown. In conclusion, this first cell reactivation phase occurs in both K59 and C15 genotypes and is characterised by an important reorganisation in cellular structure with significant increases in both nuclear- (× 40) and nucleolar-(× 67) volumes (Fig. 1J) as compared to d0 explants. Cells in this first phase of cell reactivation are considered as reactivating cells (RC).

Further examination revealed that only the responsive genotype K59 was capable of undergoing the second phase of cell reactivation (Fig. 1D). In this phase, chloroplasts are absent or, if present, have become disorganised and some organelles appeared to be fused together. The centrally-positioned nucleus increased in volume (Fig. 1J) and contained a large unique, (or occasionally double), nucleolus. Cytoplasmic strands became associated with the nucleus and the vacuole fragmented. The second cell reactivation phase is therefore characterised by profound modifications to the structure of organelles and different cell compartments, together with increases in both nuclear- (× 1.5) and nucleolar- (× 1.9) volumes as compared to the first cell reactivation phase. Our results also showed that second phase reactivated cells are able to re-enter the cell-cycle and divide (Fig. 1E) via a segmentation pathway and can therefore be considered as Fully Reactivated Cells (FRCs) according to the definition of Verdeil et al. [3]. Daughter-cells first appeared within the original volume of the FRC and gave rise to a multi-cellular somatic embryo (Fig. 1F).

These results demonstrate that K59 and C15 genotypes show important differences in their capacity to undergo cell reactivation. While K59 is able to complete the 2 cell reactivation phases, C15 cells remain blocked in the first phase. In the latter genotype, no FRCs are formed, and mitoses and multi-cellular structures are absent at d11.

#### Somatic embryogenesis (SE) but not cell reactivation (CR) is blocked in the K59 genotype by β-Glc Y

**Figure 2:**
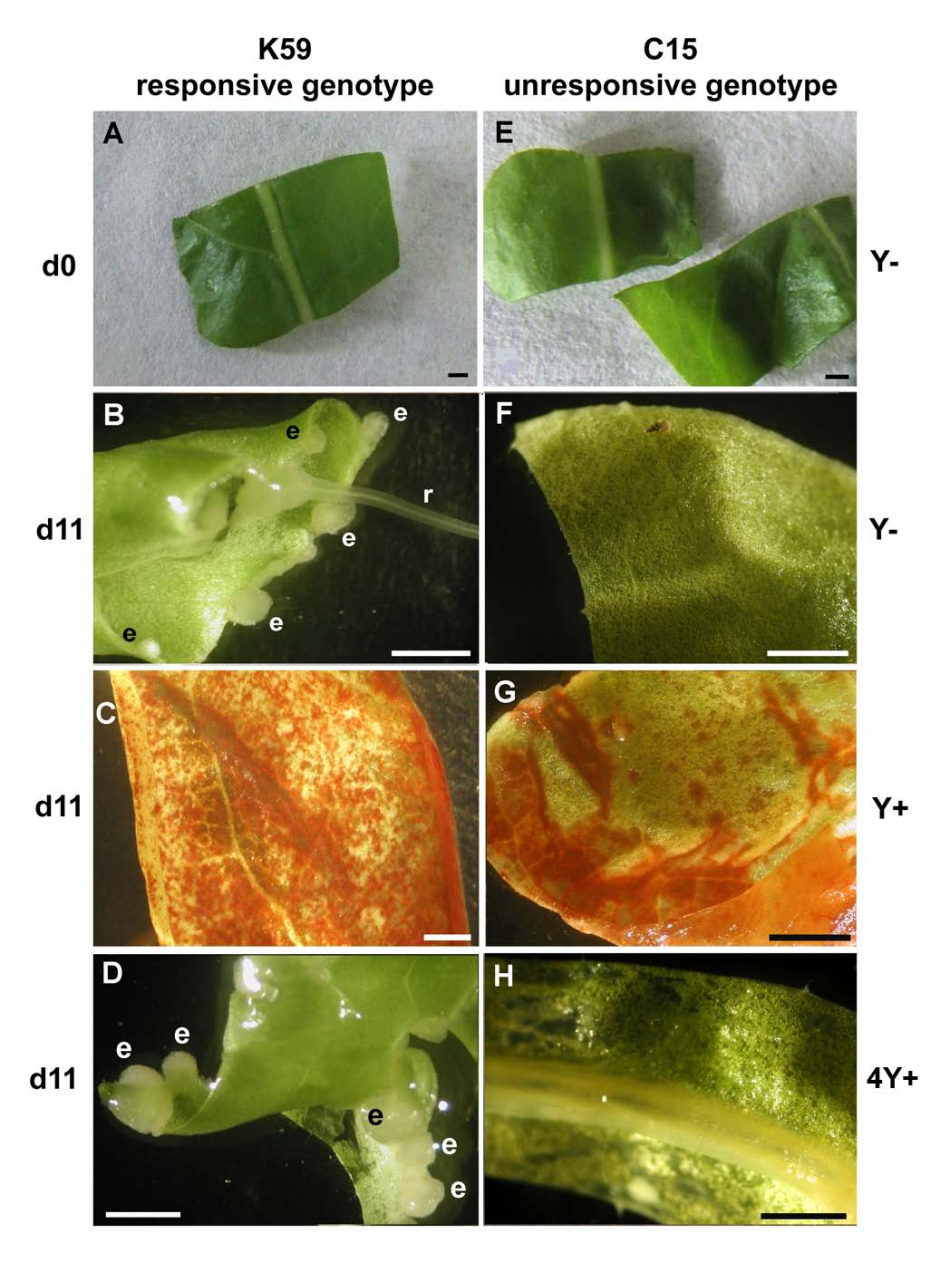
**Characterisation of phenotypes observed in chicory leaf explants from the responsive K59 genotype and the non-responsive C15 genotype, cultured in absence (control condition Y-), or in presence (Y+) of β-Glc Y**. A: Leaf explants of K59 at d0. B: Leaf explants of K59 at d11,Y-. Somatic embryos (e) can be observed at explant borders. Roots (r) can be observed during embryo development. C: Leaf explants of K59 at d11,Y+. Yariv reagent penetrates within the leaf blade. No morphogenetic pattern can be detected. D: leaf explants of K59 cultured for 4 days in presence of β-Glc Y and transferred to new medium deprived of β-Glc Y during 7 days (d11, 4Y+). Cut explant borders curl upwards and somatic embryos (e) can be observed. E: Leaf explants of C15 at d0. F: Leaf explants of C15 at d11,Y-. No morphogenetic pattern can be detected. G: Leaf explants of C15 at d11,Y+. Yariv reagent penetrates within the leaf blade. No morphogenetic pattern can be observed. H: leaf explants of C15 cultured for 4 days in presence of β-Glc Y and transferred to a new medium deprived of β-Glc Y during 7 days (d11, 4Y+). No somatic embryos can be detected. Bar = 2 mm.

We had previously demonstrated that SE in chicory roots was blocked by treatment with β-Glc Y suggesting that this reagent represents a useful tool for investigating SE in this species. In order to see whether β-Glc Y also blocked SE in the K59 embryogenic genotype we cultured leaf explants for 11 days in SE induction medium containing β-Glc Y (d11,Y+ samples). Control samples (d11,Y-) were cultured for 11 days in SE medium with no β-Glc Y. The appearance of K59 leaf explants cultured for 11 days in the absence of β-Glc Y (d11,Y-) is shown in fig. 2B. At this stage explants appeared as curled-up green fragments (Fig. 2B) and somatic embryos could be observed as white structures on the cut borders. Closer examination (data not shown) indicated that different embryo developmental stages (globular, heart and cotyledon) could be identified. In contrast, K59 genotype leaf explants cultured for 11 days in the presence of β-Glc Y (d11,Y+) do not produce somatic embryos (Fig. 2C). In these explants β-Glc Y was visible as red dots within the leaf blade. These results clearly indicate that β-Glc Y is able to block SE in the K59 genotype.

In order to see whether β-Glc Y inhibition of SE is permanent we cultured explants for 4 days in SE induction medium containing β-Glc Y, followed by transfer to medium lacking β-Glc Y for a further 7 days (d11,4Y+ samples). Our results (Fig. 2D) show that at 11 days the red dots (β-Glc Y) had disappeared and that somatic embryos were visible. Further observations (data not shown) indicated that the embryos appeared very rapidly, (after 1-2 days) following transfer to medium lacking β-Glc Y. These results indicate that β-Glc Y inhibition of SE is reversible in the K59 genotype. Identical treatments with the C15 genotype (Figs. 2F, 2G, and 2H) confirmed that this genotype is not capable of producing somatic embryos in the conditions examined.

Taken together, these results confirmed 1) that the K59 genotype, but not the C15 genotype, was responsive (embryogenic) when leaf explants were cultured in SE induction medium and 2) that the 11-day β-Glc Y-treatment blocked the *in vitro *morphogenetic development in the K59 chicory genotype. Our results also show that the inhibitory effect of β-Glc Y on SE in K59 leaf explants is not permanent and can be rapidly reversed as was previously observed in root explants of the chicory hybrid '474' [1]. Interestingly, reversible inhibition by β-Glc Y has also been reported in the case of pollen tube growth in several species [30], as well as in zygotic embryo differentiation in *Arabidopsis *[32].

**Figure 3:**
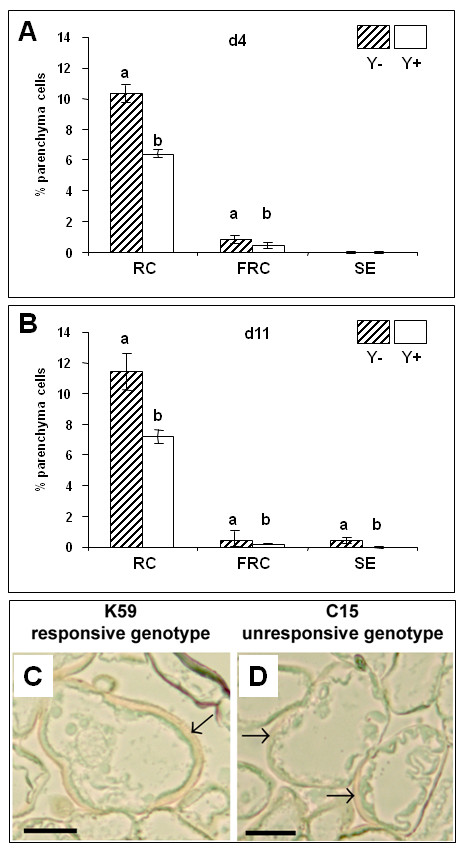
**Effects of the β-1,3-D Yariv treatment during SE induction**. A and B: Effects of β-1,3-D Yariv treatment on the occurrence (white columns) of reactivating cells (RC), fully reactivated cells (FRC) and somatic embryos (SE) in K59 explants at d4 and d11. All RC are not able to go through the second phase of cell reactivation to reach FRC status, however, the majority of FRC will form somatic embryos at d11. When β-1,3-D Yariv is applied, the numbers of RC and FRC are significantly decreased, and no somatic embryos are produced at d11. C and D: The Yariv reagent is clearly detected as an orange coloration within leaf tissue and it can be observed surrounding reactivating cells (arrows). Three micrometer semi-thin sections observed under Nomarski phase contrast. Bar = 20 μm. Hatched columns = control conditions (absence of β-1,3-D Yariv). The Student-Newman-Keuls test was applied on data collected from 10 independent slides. For one event, different letters indicate significant differences. Bars represent standard errors.

Our results indicated that SE in the K59 genotype was reversibly blocked by β-Glc Y. In order to see whether this inhibition concerned cell reactivation and/or the subsequent morphogenetic pathway we analyzed sections of leaf explants cultured for 4 days in SE induction medium containing β-Glc Y. Our results (Figs. 3A, B) showed that although β-Glc Y had a significant effect on cell reactivation (significant reduction in the percentages of RCs and FRCs), it did not completely block this process in the K59 genotype. Since no somatic embryos can be observed in explants cultured for 11 days in the presence of β-Glc Y, this would suggest that the main inhibitory effect of this reagent acts on the morphogenetic pathway following cell reactivation. The observation (data not shown) that embryos are rapidly formed following β-Glc Y removal could suggest that cells remain blocked at the FRC stage thereby allowing rapid morphogenetic development once the inhibitor is eliminated. Similar analyses of C15 genotype explants indicated that only RCs were present in both the presence and absence of β-Glc Y (data not shown).

#### SE inhibition by β-Glc Y is potentially associated with cell wall modifications

Observations of sections from leaf explants (K15 and C15 genotypes) cultured for 4 days in the presence of β-Glc Y indicated that the Yariv reagent was present as an orange coloration surrounding certain mesophyll cells (Figs. 3C, D). No coloration was observed inside these cells. Such an observation is logical since the β-Glc Y molecule is small enough to penetrate the intercellular space of plant tissues, but too large to cross the plasma membrane [26]. Since β-Glc Y is known to interact with AGPs [23,18,25] our results could suggest that β-Glc Y co-localised with cell wall AGPs.

Visual inspection of mesophyll cell walls coloured orange by β-Glc Y suggested that they were thicker than the walls of neighbouring mesophyll cells not coloured by β-Glc Y (data not shown). Interestingly, such cell wall thickening in the presence of β-Glc Y was also observed during cell reactivation in the chicory '474' hybrid [13]. In this case, further studies [33] indicated that callose was deposited around FRCs. These results could suggest that the β-Glc Y reagent modifies cell wall assembly in chicory, possibly by preventing the incorporation of secreted material into the cell wall as previously observed in lilly [34]. In this species, β-Glc Y binds to AGPs and destabilizes cell wall assembly by blocking the deposition of pectin material in the cell wall. In addition, the periplasmic space expands as a result of the accumulation of vesicles containing AGPs and pectins, and synthesis of callose is simultaneously induced. A direct link between AGPs and pectin has also been previously observed in carrot [35] where it has been shown that cell wall AGPs may be covalently linked to pectin containing a homogalacturonan structural element.

### 2. Transcriptome profiling during cell reactivation in chicory

#### Transcriptome profiling identifies genes differentially regulated between embryogenic and non-embryogenic genotypes

Our detailed microscopic analyses had shown that while both K59 and C15 genotypes undergo the first phase of cell reactivation, only K59 undergoes the second phase, followed by the formation of somatic embryos. In order to learn more about the molecular bases of these biological processes we performed transcriptome profiling using chicory specific microarrays corresponding to 1,098 genes previously identified in a chicory SSH library [2].

**Figure 4:**
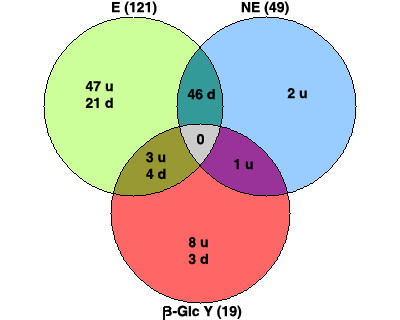
**Venn diagram depicting gene expression profiles in K59 and C15 genotypes following SE induction**. The circle E (embryogenic) represents the number of genes specifically expressed in K59 genotype (up-regulated or down-regulated in contrast with C15) or differentially expressed (with opposite profiles in K59 and C15) after 4 days of *in vitro *induction of SE (K59 d4 *vs*. K59 d0). The circle NE (non-embryogenic) represents the number of genes specifically or differentially expressed in the C15 genotype after 4 days of *in vitro *induction of SE (C15 d4 *vs*. C15 d0). The β-Glc Y ring represents the number of genes (in both K59 and C15 genotypes) whose expression is modified after 4 days of *in vitro *induction in presence of β-Glc Y (K59 or C15 4 d *in vitro *induction in presence of β-GlcY *vs*. K59 or C15 4 d *in vitro *induction in absence of β-GlcY). u: up-regulated; d: down-regulated.

In a first experiment we compared the expression profiles of d4 leaf explants under SE induction conditions *versus *d0 for both genotypes. Expression profiles are represented as a Venn diagram in Fig. 4 (circle E: K59 embryogenic genotype, circle NE: C15 non-embryogenic genotype). Seventy-five (68+7) out of the 1,098 genes analysed were found specifically or differentially expressed in E, 3 (2+1) genes were found specifically regulated in NE, and 46 genes were similarly regulated in both E and NE genotypes. These results suggest that the 78 (75+3) genes specifically or differentially regulated in the K59 and C15 genotypes during *in vitro *induction of SE could be involved in the different cell fate and SE pathways taken by these 2 genotypes. It is possible that the group of 46 genes regulated similarly in both genotypes might represent a common stress response to the induction culture conditions and/or these genes might be involved in the cell reactivation process common to both genotypes.

#### Transcriptome profiling and β-Glc Y-treatment identifies genes potentially involved in the SE morphogenetic pathway

**Figure 5:**
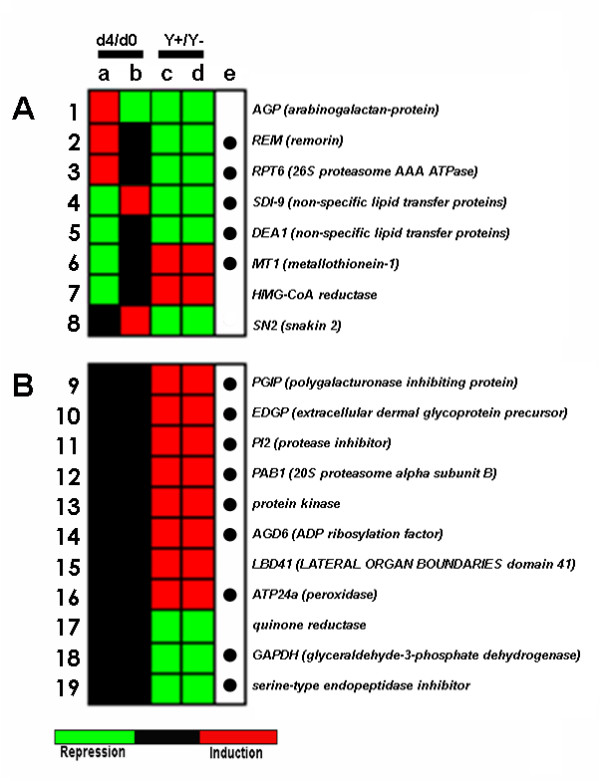
**Ternary clusters representing expression profiles of genes affected by β-GlcY-treatment**. A: genes specifically or differentially expressed in K59 and C15 genotypes, and whose expression is affected by β-GlcY; B: genes whose expression is affected by β-GlcY-treatment but is not modified during cell reactivation in K59 and C15 genotypes. Columns represents K59 d4 *vs*. K59 d0 (a); C15 d4 *vs*. C15 d0 (b); K59 d4 in presence of β-GlcY *vs*. K59 d4 in absence of β-GlcY (c); C15 d4 in presence of β-GlcY *vs*. C15 d4 in absence of β-GlcY (d). Red colour indicates gene induction, whereas green colour indicates repression. Induction corresponds to a log2 ratio ≥1, and repression to a log2 ratio ≤ -1. The column (e) corresponds to chicory genes (black circle) that have previously been described in the literature as being associated with wound-/pathogen responses in *Arabidopsis thaliana*.

Our cytological analyses (Figs. 1, 3) indicated that β-Glc Y-treatment inhibits the morphogenetic pathway leading to somatic embryo formation but has little effect on the cell reactivation process. The Yariv reagent therefore represents a useful tool to separate cell reactivation events from the SE pathway. In order to specifically identify genes potentially involved in these different processes we performed transcriptome profiling on both K59 and C15 leaf explants cultured for 4 days in the presence/absence of β-Glc Y (Figs. 4, 5). An initial examination of our results had shown that the expression profiles of d0 K59- and C15-explants did not show any significant differences (see additional file 2), thereby allowing a direct comparison of the results obtained separately (d4 samples, presence/absence β-Glc Y) for both genotypes.

Our results (Fig. 4, circle β-Glc Y) indicate that only relatively few genes (19 from 1,098) show changes in gene expression in the presence of β-Glc Y. Of these 19 genes, 8 (7 + 1) genes were both differentially expressed between K59 and C15 during SE induction and affected by β-Glc Y-treatment. These results suggest that these genes could be involved in the morphogenetic pathway leading to somatic embryo formation in chicory. Among the 8 genes, 2 encode cell wall proteins: *arabinogalactan-protein *(DT212818), and *remorin *(DT211027). The other 6 genes are not directly related to the cell wall-compartment and include *26 S proteasome AAA ATPase subunit 6 *(DT212545), *non-specific lipid transfer proteins *(DT213069, DT212585), *metallothionein-1 *(DT211058), *HMG-CoA reductase *(DT213261), and *snakin 2 *(DT211070) (Fig. 5A).

The gene encoding the peptide core of a putative AGP (DT212818) was differentially expressed in K59 (up-regulated) and C15 (down-regulated) genotypes during SE induction, and repressed (both genotypes) by β-Glc Y-treatment (Fig. 5A line 1). Such an observation is in agreement with our previous results in chicory [2], as well as Affymetrix transcriptome analyses in *Arabidopsis *cells [36] showing that the expression levels of 9 out of the 43 genes encoding an AGP core polypeptide were modified by β-Glc Y treatment. In *Arabidopsis *cells, no AGP gene was induced more than 2-fold following 10 h β-Glc Y-treatment but several AGPs were down-regulated more than 2-fold. These results, together with the observations [1,13,20-22] that AGPs are associated with the cell re-differentiation process in both carrot and chicory provide strong support for the role of the putative AGP (DT212818) in chicory SE. Only one AGP-like gene (DT212818, corresponding to contig 0687) was represented on our microarray design since it was the only one whose differential expression was confirmed by Q-RT-PCR [2]. Nevertheless, it would obviously be interesting to investigate the effect of β-Glc Y-treatment on the expression of other chicory AGP genes.

Our results (Fig. 5A, line 2) also showed that a second 'cell wall gene' *REM*, a remorin encoding gene (DT211027), was specifically up-regulated during SE induction in K59 cells, but not in C15 cells. The gene was also down-regulated by β-Glc Y treatment in both genotypes. Remorins were first discovered in a screen for plasma membrane proteins differentially phosphorylated in the presence of oligogalacturonides [37] and were recently reviewed [38]. The observation that a chicory *REM *(DT211027) was down-regulated by β-Glc Y could be related to the fact that such treatment immobilises oligogalacturonic acids (OGAs) and polygalacturonic acids (PGAs) in the periplasmic space [34]. Our results showed that both *REM *(DT211027) and *AGP *(DT212818) were up-regulated in the K59 genotype during SE induction. These observations suggest that cell wall reorganization could play an important role in SE, with AGP potentially contributing to the transport of pectin-like material [34] or covalent linkages [35], and remorin being involved in OGA-/PGA-binding and signalling [39]. Interestingly, we have previously shown that SE in chicory involves a complete re-organisation of cell wall architecture and pectin modifications [40].

Of the other 'non-cell wall genes' potentially involved in SE, *RPT6 *encoding the regulatory AAA ATPase subunit 6 of the 26 S proteasome, was up-regulated during the induction phase in K59, but not C15 (Fig. 5A line 3). β-Glc Y-treatment considerably down-regulated *RPT6*-expression in both genotypes. The proteasome pathway is one of the most elaborate regulatory mechanisms in plants [41] and has been shown to be involved in plant development. For example MG132, a specific inhibitor of the 26 S proteasome, interferes with the entry of plant cells into the S phase of the cell cycle [5] as well as the passage G2/metaphase in yeast [42]. The observation that *RPT6*-expression is genotype-dependant and modified by β-Glc Y-treatment in chicory could suggest that the responsiveness to the induction of *in vitro *morphogenesis might be proteasome-mediated. In this case, it is possible that RPT6 could influence the transition from RC to FRC status and, consequently, cell fate determination in chicory explants.

Our results (Fig. 5A, lines 4 and 5) also showed that two genes encoding nsLTPs (DT213069, DT212585) were differentially expressed between K59 and C15 during SE induction. β-GlcY-treatment down-regulated the expression of these 2 genes in both genotypes. The first gene (DT213069) shares sequence homology with *SDI-9 *from *Helianthus annuus *and the second gene (DT212585) shares sequence homology with *DEA1 *from *Lycopersicon esculentum*. The presence of nsLTPs during SE has previously been reported in other species such as grapevine [43,44] and carrot [45]. In the latter species, it was shown [46] that nsLTP gene expression followed, rather than preceded SE. Interestingly, we have previously shown [47] that a 9-kDa acidic nsLTP-like protein was secreted by the embryogenic cells of roots in the chicory interspecific hybrid '474' and that SE in chicory is also associated with increases in phosphatidylcholine and triacylglycerols thereby suggesting that changes in lipid and fatty acid composition might be involved in this process [48].

Another gene, *Metallothionein-1 *(*MT1*) was down-regulated in K59 but not C15 during *in vitro *induction, and up-regulated in both genotypes by β-GlcY (Fig. 5A, line 6). The described role of metallothioneins is to bind metals and these proteins are therefore involved in plant responses to salinity and oxidative stress [49]. Alterations in the cellular redox status have been shown to be associated with *in vitro *SE [50-57]. In chicory, glutathione S-tranferases (DT212623 and DT213268) that belong to a complex anti-oxidant mechanism within the cell, together with catalase (DT213897) and MT1 (DT213289) were found to be more abundant or exclusively present in a non-embryogenic subtractive library [2]. Such an observation could suggest that 'low' levels of oxidative stress are associated with a non-embryogenic status in chicory and that 'higher' levels are associated with SE events. The fact that MT1 was down-regulated in K59 during SE induction is in agreement with this hypothesis. In this case it is possible that the up-regulation of MT1 observed during β-GlcY-treatment not only protects the cells against oxidative stress, but also modifies the cellular redox status thereby impeding SE formation.

Finally, two other genes with a less evident role in SE events were also differentially regulated between the two genotypes during SE induction. *3-hydroxy-3-methylglutaryl-CoA reductase *(*HMG-CoA reductase*) encodes an enzyme in the mevalonate pathway [58] and our results (Fig. 5A, line 7) showed that the corresponding chicory gene (DT213261) was down-regulated in the embryogenic genotype K59 during *in vitro *induction. Such an observation is interesting since we had previously shown that the corresponding transcript was abundant in a non-embryogenic subtractive library [2]. The fact that β-Glc Y-treatment up-regulated the *HMG-CoA reductase *gene in both chicory genotypes also suggests that the expression of this gene is negatively correlated with SE. The last differentially-regulated gene *Snakin 2 *(*SN2*) is putatively involved in developmental regulation processes and in defence [59,60]. Our results (Fig. 5A, line 8) showed that the gene (DT211070) was up-regulated in C15, but not K59, during *in vitro *SE induction. β-Glc Y-treatment down-regulated *SN2 *gene expression in both genotypes.

##### β-Glc Y-treatment affects the expression profiles of genes not involved in SE

Our results showed that β-Glc Y-treatment modified the expression profiles of 11 genes not involved in SE (i.e. genes showing no differential expression between d0 and d4 in both genotypes; Figs. 4 and 5B). These genes encode polygalacturonase inhibiting protein (PGIP, DT213313), extracellular dermal glycoprotein precursor (EDGP, DT213213), protease inhibitor (PI2, DT210863), 20 S proteasome alpha subunit B (PAB1, N102_E10), protein kinase family protein (DT212527), ADP ribosylation factor GTPase activating protein (AGD6, DT213194), LATERAL ORGAN BOUNDARIES domain protein 41 (LBD41, DT213326), peroxidase (ATP24a, DT212627), quinone reductase (DT213653), glyceraldehyde-3-phosphate dehydrogenase (GAPDH, DT212581), and serine-type endopeptidase inhibitor (DT213247).

The observed modifications in the expression profiles of these genes can be most likely interpreted as a direct or indirect response of plant cells to β-Glc Y independent of SE-related events. For example, PGIP (Fig. 5, line 9) is a secreted protein involved in plant defence at the cell wall [61] where it is believed to modulate the activities of fungal polygalacturonases targeting cell wall pectins [62]. In lilly, β-Glc Y has been shown [34] to block pectin-containing vesicules in the periplasmic space and it is therefore possible that the exposure of explants to this reagent interferes with correct cell wall assembly thereby provoking a defence response involving PGIP gene up-regulation. Similarly, a chicory homologue (DT213194) of *Arabidopsis *AGD6, an ADP-ribosylation factor GTPase activating protein (ArfGAP) was up-regulated during β-Glc Y-treatment (Fig. 5 line 14). *Arabidopsis *contains 15 proteins with ArfGAP domains termed AGD proteins [63]. The GTP-bound form of ARF is essential for the maintenance of normal Golgi morphology [64] and correct cell wall assembly [65]. It is therefore possible that β-Glc Y interferes with correct cell wall assembly thereby provoking modifications in a gene (DT213194) involved in this process.

##### β-Glc Y treatment mimics wound- and pathogenic-like effects

Previous studies [36,66] have shown that β-Glc Y-treatment induces a wound response in *Arabidopsis *cells including modifications in gene expression. Similarly, comparative studies of wound and stress signalling [67] have revealed the existence of extensive cross-talk in *Arabidopsis *and other species. Interestingly, a number of reported mimetic effects between the response to β-Glc Y and the responses to wounding and pathogen attack in chicory have also been reported [36,59,61,66,68-72]. Examination of our results (Fig. 5, column e) showed that, of the 19 genes whose expression was modified by β-Glc Y, 14 also showed similar modifications to wounding or pathogen treatment. Such an observation suggests that β-Glc Y-treatment in chicory mimics a wounding-/pathogen-response. In other plants, β-Glc Y-treatment has been shown to induce different effects depending upon the explant type, culture medium and species. For example, in rose cell suspension cultures, a 7-day β-Glc Y-treatment did not lead to a loss of viability [26], whereas in *Arabidopsis *cells, β-Glc Y-treatment led to loss of viability, and programmed cell death [36]. In chicory, we did not observe any necrosis or collapsed cells in either macroscopic or microscopic leaf samples suggesting that the 'wounding' effect of β-Glc Y-treatment in our system remains relatively mild despite the modifications observed in 'defence' gene expression profiles.

## Conclusions

The two analysed chicory genotypes differed in their morphogenetic capacities. The C15 unresponsive (non-embryogenic) genotype was able to enter the first phase of cell reactivation, but not the second phase, and was therefore unable to express any morphogenetic pathway. In contrast, the K59 responsive (embryogenic) genotype was able to enter both the first and second phases of cell reactivation and was therefore able to undergo SE. β-Glc Y had little effect on the first phase of cell reactivation but proved to be a successful tool to reversibly block SE in the K59 genotype. The use of this reagent together with transcriptome profiling allowed us to identify 8 genes that are potentially involved in the second phase of cell reactivation and/or SE in chicory explants. Two (*AGP*, *REM*) of the 8 genes were associated with cell wall activities underlying the importance of this cellular compartment in developmental processes. Overall, this study allowed us to obtain a detailed description of cell reactivation events in chicory at the cellular and transcriptomic levels and to identify key genes potentially involved in this complex process.

## Methods

### Plant material

Two chicory (*Cichorium intybus *L.) genotypes were used: K59, a 'responsive' (embryogenic) genotype identified on the basis of its capacity to undergo SE, and C15, a 'non-responsive' (non-embryogenic) genotype [2]. Both genotypes belong to the Hungarian landrace Koospol (genetic resource, Ets. Florimond Desprez, Cappelle en Pévèle, France). The C15 genotype was obtained by self pollination of K59, and therefore shares a common genetic background with the latter genotype. Leaf explants obtained from greenhouse plants were cultured in an organogenesis medium [2]. Plantlets were then sub-cultured *in vitro *on solid Heller medium in glass tubes [73].

### SE induction and β-Glc Y-treatment

Leaf explants (approximately 1 cm^2^) were excised from 2-month-old *in vitro *plantlets (K59 or C15 genotypes). Explants (d0 sample) were then 1) frozen in liquid nitrogen for subsequent RNA extraction, 2) fixed for subsequent microscopy, or 3) cultured for 4 days in SE induction medium. For 3), six explants were placed in 20 mL SE induction medium [7] containing either 250 μM β-Glc Y (Y+: treated explants), or no β-Glc Y (Y-: control explants), as described in Chapman *et al. *[1]. β-Glc Y was synthesised from phloroglucinol and p-aminophenyl-D-glycopyranoside precursors (Sigma) according to the method of Yariv *et al. *[74]. Twelve plants of each genotype were used per condition (Y-, Y+).

Explants were cultured on an orbital shaker for 4 days at 35°C in obscurity. After 4 days culture, explants (d4,Y+ or d4,Y-) were 1) frozen in liquid nitrogen for subsequent RNA extraction, 2) fixed for subsequent microscopy, or 3) transferred into new induction medium containing no β-Glc Y and cultured for a further 7 days giving rise to a total culture period of 11 days.

At the end of the 11-day culture period, explants (d11,4Y+ and d11,4Y-) were fixed and examined by microscopy in order to determine the nature of the expressed morphogenetic pathway (if any). Explants (d11,Y+) were also cultured for 11 days in SE induction medium containing either 250 μM β-Glc Y (d11,Y+ samples), or no β-Glc Y (d11,Y- samples).

The β-Glc Y analogs, β-D-mannan Yariv phenylglycoside (β-Man Y) or α-D-galactosyl Yariv (α-Gal Y) are usually used as negative controls in experiments involving β-Glc Y-treatments. However, we did not use either of these controls as we had previously shown [1], (D. Windels, unpublished data), that β-Man Y did not cause any noticeable cytological or morphological modifications in chicory root- or leaf-explants. In addition, a previous detailed study in *Arabidopsis *[36] indicated that β-Man Y induced changes in the expression of 64 genes. The chicory microarrays used in the current study did not include cDNAs corresponding to the genes affected by β-Man Y in *A. thaliana*.

### Histology

Samples (d0, d4 and d11) were fixed in a formaldehyde: acetic acid: ethanol solution (FAE, 3.5:6.5:90, *v/v*.), progressively dehydrated in an alcohol series and infiltrated with Technovit 7100 resin (Kulzer). Sections (3 μm) were cut using a Leica RM 2065 microtome and stained with a 0.5% (w/v) aqueous solution of Toluidine blue O (TBO) (Sigma) for examining cell reorganisation. Some sections were directly observed without any staining to detect the presence of β-GlcY which appears as an orange labelling under brightfield microscopy. Sections were examined with a Leica DM2000 microscope coupled to a Leica DFC320 camera. Images were analysed with the Leica Application Suite program.

Three embedded leaf fragments collected from three *in vitro *plantlets were analyzed per sample. Counting was performed on 200 independent slides; serial sections (12-15 per slide) were obtained from zones separated by at least 3 cell layers (approximately 150 μm) in order to observe different regions of one embedded leaf fragment. This method allows us to determine (i) the shape and volume of the nucleus and nucleolus, (ii) the cell fate in three dimensions, and (iii) the size of multi-cellular structures. Data were collected from the total length of a transversal section then recalculated for 100 parenchyma cells. Statistical tests were done according to the Student-Newman-Keuls method (*P *< 0.05).

### RNA extraction

Total RNA was extracted from d0, d4 control (Y-) and d4 treated (Y+) explants using a Tri reagent kit (Euromedex) and purified with the RNeasy MinElute kit (Qiagen). RNA integrity was checked by capillary electrophoresis (Agilent 2100 Bioanalyser, Agilent technologies).

### Microarray production

Annotated chicory ESTs [2], representing 1,098 unique genes, were used to produce microarrays. These ESTs have been incorporated in the CGPD (Compositae Genome Project Database) [75]. Six microliters of bacterial culture stored with glycerol were put into 100 μl TTE buffer (1% triton × 100, 20 mM Tris-HCl, 2 mM EDTA, pH 8.0) in 96 well plates. Plates were incubated 10 min at 95°C and spun at 1,200 g for 5 min. The cDNA inserts were amplified using aminated adaptator-specific forward primer NP1 and reverse primer NP2 (Clontech) flanking the cDNA insert. For each clone, 3 independent reactions were carried out in a volume of 100 μl containing 1× ThermoPol Detergent Free Reaction Buffer (Biolabs), 2 mM MgCl2, 0.25 mM of each dNTP, 0.25 μM of each primer, 2.5 U of Taq DNA Polymerase (Biolabs) and 7 μl of DNA template in TTE buffer. The PCR reaction was performed for 3 min at 94°C, 35 cycles at 94°C for 30 s, 60°C for 30 s, and 70°C for 90 s, and 70°C for an additional 10 min. The 3 PCR products were mixed and purified using the Multiscreen-PCR Kit (Millipore), analyzed by gel electrophoresis, and quantified by OD measurement at 260 nm. The DNA was dried in a Speed Vac (Savant Instruments), and redissolved in spotting solution (0.1 M MES, 20% DMSO) to obtain a final concentration of 0.5 μg/μl. Preparation and hybridization of cDNA microarrays were performed according to Hot *et al. *[76] except that PCR fragments were spotted three times onto 25×75 mm Codelink Activated Slides (Amersham) using the Affymetrix 417 Arrayer. Spotted slides were treated with blocking solution (50 mM Trizmahydrocloride, 50 mM Tris(hydroxymethyl)aminomethane, 0.3% ethanolamine, pH 9), containing 0.1% SDS at 50°C for 30 min. Slides were washed twice in distilled water for 2 min at room temperature, once in SSC 4× 0.1% SDS for 60 min at 50°C, once in distilled water for 1 min at room temperature, once in distilled water for 2 min at 95°C, and finally twice in distilled water for 2 min at room temperature, before being dried by centrifugation for 5 min at 180 g.

### Transcriptome analyses

Ten μg of total RNA were used for each experiment. Reverse transcription and fluorochrome incorporation were performed in a MJ PTC-200 Peltier Thermal Cycler, using oligo-dT (Roche), the Superscript II Kit (Invitrogen) and CY3- and CY5-labelled d-CTP (Amersham). The QIAquick PCR Purification Kit (Qiagen) was used for target purification prior to 14-18 h hybridization (DigEasy hybridization buffer; Roche) at 42°C in a Corning chamber. Slides were washed successively in 4 ×, 2 × (with SDS 0.1 ×), 0.2 ×, and 0.1 × SSC.

For both genotypes (K59, C15), gene expression profiles were firstly compared between d0 samples (non-induced) and d4 samples (induced) cultured in SE induction medium in the absence of β-GlcY (samples Y-). Gene expression profiles were then compared between d4 samples cultured in the absence of β-GlcY (samples Y-) and in the presence of β-GlcY (samples Y+). In order to compare expression profiles from both genotypes, direct comparison was performed on d0 samples (K59, C15). Three biological replicates were used for each analysed condition. Dye labelling for each paired sample was reversed in two subsequent individual hybridizations giving rise to a total of six hybridizations per condition.

Images were acquired with a GenePix4000B scanner (Molecular Devices, Sunnyvale, CA, USA) and analyzed with GenePix Pro 6.0 software (Axon). Artefactual, saturated, or low-signal spots were eliminated from the analysis and the background subtracted median intensities used for calculations. For each slide a global lowess followed by a print-tip median normalization was performed using R packages [77] as implemented in Goulphar [78]. In order to exclude possible dye effects, all probes exhibiting an opposite behaviour in the swapped-dye experiment were eliminated from the final analysis. To identify genes displaying a change in expression over repetitions, a script utilizing library functions in R with a false discovery rate (FDR) of less than 5% was used for all experimental conditions. Only genes with smooth expression profiles were retained. The SAM [79] was used to identify differentially expressed genes over different conditions and log2(ratio) ≥1 and ≤ -1 were used for filtering gene expression profiles. The Venn Diagram Generator [80] was used to create Venn diagrams. Hierarchical clustering analysis used the Treeview program [81]. The significant transcriptome data are available in additional file 2. All the microarray data have also been submitted to the Gene Expression Omnibus (GEO) database [82]. The accession number is GSE15502.

### Quantitative RT-PCR

Labelling for quantitative RT-PCR was performed with iScript cDNA synthesis kit (Bio-Rad). Primer design (see additional file 3) and quantitative RT-PCR was performed as described in Legrand *et al. *[2]. Relative quantification was performed using the 2^-ΔΔCT ^method [83]. Three biological repetitions and two technical repetitions were done for each experiment. The expression patterns of 6 selected genes that had shown differential expressions in microarray analyses were verified by Q-RT-PCR (see additional file 4). Student t-test was applied on data collected from Q-RT-PCR and microarray analyses. For all comparisons, t values for 0.005 confidence threshold indicated that differences were not significant.

## Abbreviations

SE: somatic embryogenesis; β-GlcY: beta-D-glucosyl Yariv reagent; AGP: arabinogalactan-proteins; E/NE: embryogenic/non-embryogenic; EST: expressed sequence tag; SSH: suppression subtractive hybridization; RC: reactivating cell; FRC: fully reactivated cell; SAM: Significance Analysis of Microarrays; FDR: false discovery rate; TBO: Toluidine blue O; SSC: sodium saline citrate; SDS: sodium dodecyl sulfate.

## Authors' contributions

ALD designed the study, performed transcriptomic analyses and biochemical experiments, contributed to the interpretation of the results and to the writing of the manuscript, and is the corresponding author. LL carried out the histological experiments, RNA extractions and microarray hybridizations, real-time RT-PCR analyses and drafted the manuscript. SL and MCQ contributed to the SSH library production, EST annotation and microarray production. LH contributed to the microarray design and production. DH and YL participated in microarray data processing and interpretation. SH participated in the interpretation of cell wall-related results and writing of the manuscript. JLH participated in planning and supervision of the study, and in reviewing the manuscript. MCQ and TH contributed to the interpretation of the results and to the writing of the original version of the manuscript. ASB contributed to the design of the study, performed and supervised all histological experiments, and interpretation of the results, and participated in the writing of the manuscript. All authors read and approved the final manuscript.

## Supplementary Material

Additional file 1 **Detailed cytological modifications occurring in cultured explants from K59 (responsive) and C15 (non responsive) chicory genotypes during cell reactivation**. Click here for file

Additional file 2 **Log2(ratio) data sets of significant transcriptome analyses. a: putative morphogenetic-related genes specifically expressed in K59 or C15 genotypes**. b: putative cell-reactivation and stress response-related genes showing common regulations in K59 and C15 genotypes. c: genes without a direct role in cell reactivation or morphogenesis and whose expression is specifically modified in response to β-Glc Y. Data columns: K59 d4 *vs*. K59 d0; C15 d4 *vs*. C15 d0; K59 4 d *in vitro *induction in presence of β-GlcY *vs*. K59 4 d *in vitro *induction in absence of β-GlcY; C15 4 d *in vitro *induction in presence of β-GlcY *vs*. C15 4 d *in vitro *induction in absence of β-GlcY; K59 d0 *vs*. C15 d0; SAM score for differentially expressed genes. Click here for file

Additional file 3 **Primer sets used for real-time RT-PCR**. Click here for file

Additional file 4 **Q-RT-PCR results compared with microarray results. A: K59 d4 *vs***. K59 d0; B: C15 d4 *vs*. C15 d0; C: K59 4 d *in vitro *induction in presence of β-GlcY *vs*. K59 4 d *in vitro *induction in absence of β-GlcY; D: C15 4 d *in vitro *induction in presence of β-GlcY *vs*. C15 4 d *in vitro *induction in absence of β-GlcY. Student t-test was applied on data collected from Q-RT-PCR and microarray analyses. For all comparisons calculated t values for 0.005 confidence threshold, indicated that differences were not significant. Click here for file
