# Supplementary material for: Identification of novel genes potentially involved in somatic embryogenesis in chicory (Cichorium intybus L.)
Source: BMC Plant Biol. 2010 Jun 22;10:122. doi: 10.1186/1471-2229-10-122 (PMC3017773; doi:10.1186/1471-2229-10-122)
Supplement: Additional file 1 — Detailed cytological modifications occurring in cultured explants from K59 (responsive) and C15 (non responsive) chicory genotypes during cell reactivation. [file 1471-2229-10-122-S1.PDF]

Additional file 1. Detailed cytological modifications occurring in cultured explants from K59 (responsive) and C15 (non responsive) chicory genotypes during cell reactivation. Measures were made on serial sections/slide, and on a total of 200 slides (corresponding to nine leaf-fragments for each genotype). N/A = not applicable, ND = not determined.

	Cytological characteristics								
Genotype/ phase	Nucleus position	Nucleus volume (μm^3), shape	Nucleolus visible ?	Nucleolus volume (μm^3)	Chloroplast No., position	Chloroplast aspect, size (µm)	Distribution cytoplasm	Aspect vacuome	Corresponding Figs.
K59/d0	Adjacent cell wall	500 ± 10 , flattened	No	N/A	14-16, Adjacent cell wall	Structured, 3 x 1.5	Thin layer adjacent to cell wall	Single large turgid vacuole	1A, 1J
C15/d0	Adjacent cell wall	500 ± 10, flattened	No	N/A	14-16, Adjacent cell wall	Structured, 3 x 1.5	Thin layer adjacent to cell wall	Single large turgid vacuole	1G, 1J
K59/ Phase 1, Early CR	Adjacent cell wall	1,558 ± 48, spherical	Yes	33.4 ± 10	6-10, adjacent cell wall and nucleus	Spherical, 3-4	Thin layer adjacent to cell wall	Single large turgid vacuole	1B, 1J
C15/ Phase 1, Early CR	Adjacent cell wall	1,469 ± 39, spherical	Yes	37 ± 9	4-7, adjacent cell wall and nucleus	Spherical, 3-4	Thin layer adjacent to cell wall	Single large turgid vacuole	1H, 1J
K59/ Phase 1, Late CR	Distant cell wall	19,151 ± 125, spherical	Yes (double)	267.9 ± 40.8	5-7, adjacent cell wall and nucleus	Disorganized, fused	Thin layer adjacent to cell wall	Single large turgid vacuole	1C, 1J
C15/ Phase 1, Late CR	Distant cell wall	18,523 ± 178, spherical	Yes	319 ± 46	5-7, adjacent cell wall and nucleus	Disorganized, fused	Thin layer adjacent to cell wall	Single large turgid vacuole	1C, 1J
K59/ Phase 2	Central	27,819 ± 325	Yes	523.3 ± 50.2	ND	Disorganized/absent	Thin layer adjacent to cell wall	Vacuole fragmented by cytoplasmic strands	1D, 1J
C15 / Phase 2	This phase is never observed in the C15 genotype								

This document was created with Win2PDF available at http://www.win2pdf.com.
The unregistered version of Win2PDF is for evaluation or non-commercial use only.
This page will not be added after purchasing Win2PDF.
