# Supplementary material for: Identification of novel genes potentially involved in somatic embryogenesis in chicory (Cichorium intybus L.)
Source: BMC Plant Biol. 2010 Jun 22;10:122. doi: 10.1186/1471-2229-10-122 (PMC3017773; doi:10.1186/1471-2229-10-122)
Supplement: Additional file 3 — Primer sets used for real-time RT-PCR. [file 1471-2229-10-122-S3.PDF]

Additional file 3 - Primer sets used for real-time RT-PCR

Gene	Accession no	Sense primer (5' - 3')	Anti-sense primer (5' - 3')
AGP	DT212818	CCCAGATGCTCACCCACTTCC	TCAAACACCACCCCTCAACTACC
REM	DT211027	CGTCGAAAGGAAGCTGGAGGAG	CTCTGGTTGATTCAGCGGATGC
MT1	DT211058	CGACGATCACGGTGGCGG	AAGTGCGGATGCGGCTCAAG
SN2	DT211070	CTCGCCACTGGTTGGAACG	GACCCACAAAACACTGCTGC
RPT6	DT212545	GAAGCATCAAATAAAATCAAGGTC	ATCAATACGGTTTGTAGCCATC
HMG-CoA reductase	DT213261	TTCTGGGCAGGGTCTTGTCC	CCTCACTGGGTCTGCGATGG
actine	DY8000534	TCACCACCACAGCCGAACG	GCAGCTTCCATTCCGATGAGAG

This document was created with Win2PDF available at http://www.win2pdf.com.
The unregistered version of Win2PDF is for evaluation or non-commercial use only.
This page will not be added after purchasing Win2PDF.
