# Supplementary material for: Identification of novel genes potentially involved in somatic embryogenesis in chicory (Cichorium intybus L.)
Source: BMC Plant Biol. 2010 Jun 22;10:122. doi: 10.1186/1471-2229-10-122 (PMC3017773; doi:10.1186/1471-2229-10-122)
Supplement: Additional file 4 — Q-RT-PCR results compared with microarray results. A: K59 d4 vs. K59 d0; B: C15 d4 vs. C15 d0; C: K59 4 d in vitro induction in presence of β-GlcY vs. K59 4 d in vitro induction in absence of β-GlcY; D: C15 4 d in vitro induction in presence of β-GlcY vs. C15 4 d in vitro induction in absence of β-GlcY. Student t-test was applied on data collected from Q-RT-PCR and microarray analyses. For all comparisons calculated t values for 0.005 confidence threshold, indicated that differences were not significant. [file 1471-2229-10-122-S4.PDF]

Additional file 4 - Q-RT-PCR results compared with microarray results.

A: K59 d4 *vs*. K59 d0; B: C15 d4 *vs*. C15 d0; C: K59 4d *in vitro* induction in presence of ß-GlcY *vs*. K59 4d *in vitro* induction in absence of ß-GlcY; D: C15 4d *in vitro* induction in presence of ß-GlcY *vs*. C15 4d *in vitro* induction in absence of ß-GlcY. Student t-test was applied on data collected from Q-RT-PCR and microarray analyses. For all comparisons calculated t values for 0.005 confidence threshold, indicated that differences were not significant.

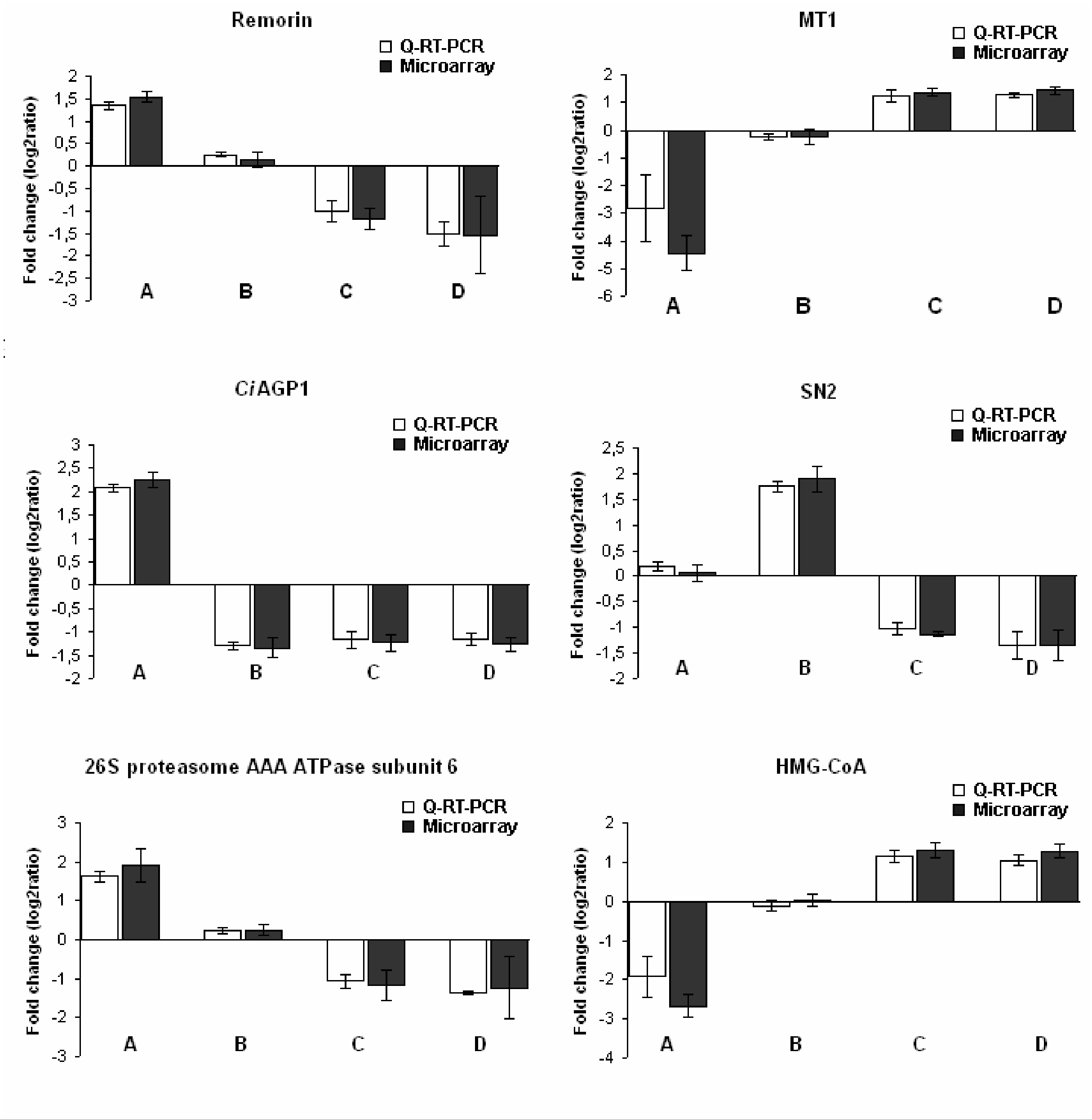

This document was created with Win2PDF available at http://www.win2pdf.com.
The unregistered version of Win2PDF is for evaluation or non-commercial use only.
This page will not be added after purchasing Win2PDF.
